# Elevated levels of hsa_circ_006100 in gastric cancer promote cell growth and metastasis via miR‐195/GPRC5A signalling

**DOI:** 10.1111/cpr.12661

**Published:** 2019-07-18

**Authors:** Min Liang, Guanqun Huang, Zhaoyu Liu, Qi Wang, Zhongjian Yu, Zhile Liu, Hai Lin, Ming Li, Xinke Zhou, Yanfang Zheng

**Affiliations:** ^1^ Department of Oncology Zhujiang Hospital of Southern Medical University Guangzhou China; ^2^ Department of Oncology the Fifth Affiliated Hospital of Guangzhou Medical University Guangzhou China; ^3^ Department of General Surgery The Fifth Affiliated Hospital of Guangzhou Medical University Guangzhou China; ^4^ Department of Center Laboratory The Fifth Affiliated Hospital of Guangzhou Medical University Guangzhou China

**Keywords:** CircRNA, gastric cancer, GPRC5A, miR‐195

## Abstract

**Objectives:**

Circular RNAs (circRNAs) are non‐coding RNAs, some of which are thought to be involved in gastric cancer development. Here, we examined the functions of circRNA hsa_circ_006100 in gastric cancer cells and an animal model of gastric cancer.

**Materials and Methods:**

The expression of hsa_circ_006100, miR‐195 and various functional genes was determined by quantitative RT‐PCR. Cell viability, clone formation, apoptosis and cell migration/invasion abilities were analysed by the CCK‐8 assay, crystal violet staining, Hoechst staining and Transwell assay, respectively. A tumour model was established by subcutaneously injecting tumour cells into nude mice. Levels of protein expression were analysed by Western blotting and immunohistochemistry.

**Results:**

A bioinformatics analysis showed that miR‐195 was negatively co‐expressed with hsa_circ_006100. Patients with a high hsa_circ_006100 level or low miR‐195 level had tumours with a high TNM stage, poor cellular differentiation and lymph node metastasis. miR‐195 was targeted and inhibited by hsa_circ_006100. Overexpression of hsa_circ_006100 enhanced cellular viability and proliferation, while miR‐195 suppressed hsa_circ_006100‐enhanced cell growth and induced apoptosis in MGC‐803 and AGS cells. Forced hsa_circ_006100 expression promoted the migration and invasion of MGC‐803 and AGS cells, while those activities were inhibited by miR‐195. Mechanistically, GPRC5A was predicted as a target of miR‐195 and was upregulated in gastric cancer. A miR‐195 inhibitor restored cell viability, proliferation, migration and invasion, and repressed apoptosis via GPRC5A. In vivo studies showed that knockdown of hsa_circ_006100 delayed tumour growth, reduced PCNA expression and upregulated miR‐195 and BCL‐2 expression which was restored by miR‐195 inhibition due to GPRC5A/EGFR signalling, and changed the EMT phenotype in vivo.

**Conclusions:**

Hsa_circ_006100 functions as an oncogene in gastric cancer and exerts its effects via miR‐195/GPRC5A signalling.

## 1 INTRODUCTION

Gastric cancer (GC) is a common malignant tumour, and despite its declining incidence over the past century, it is a major cause of cancer‐related death worldwide. In Eastern Asia, the GC mortality rate ranks third among all cancer types.^1^ GC originates from superficial mucosal epithelial cells of glands in the stomach and usually manifests as adenocarcinoma.^2^ Despite the great progress being made in cancer therapy, the survival rates of GC patients have remained dismal, largely because the disease is usually diagnosed at an advanced stage when extensive tissue invasion and distant metastasis have already occurred. In most areas of the world, the overall 5‐year survival rate of GC patients is <25%. The development and occurrence of GC metastases are regulated by a variety of oncogenes and tumour suppressor genes, and this method of regulation might help facilitate the early diagnosis and treatment of gastric cancer.^3^, ^4^

Non‐coding RNAs (ncRNAs) are distinguished by their sizes, which range from short RNAs (including microRNAs) to long RNAs that span up to hundreds of kbs (>200 nt), such as long non‐coding RNA (LncRNAs) and circular RNAs (circRNAs).^5^ Through their interactions with DNA, RNA, and proteins, ncRNAs influence protein stability and various gen regulatory activities, including chromatin remodelling, transcription and mRNA translation during many physiological and pathological processes, such as carcinogenesis.^6^, ^7^ We previously identified a series of ncRNAs involved in the N‐methyl‐N'‐nitro‐N‐nitrosoguanidine (MNNG)‐induced transformation of GES‐1‐T human gastric epithelial cells. One of those ncRNAs (LncRNA LOC101927497) functions as a tumour suppressor by interacting with miR‐574 to inhibit GC cell proliferation and migration, suggesting that ncRNAs might assist in regulating GC progression.^8^

CircRNAs are newly discovered endogenous ncRNAs that are hundreds or even thousands of bases in length and share a covalently closed structure. CircRNAs exhibit tissue‐ and developmental stage‐specific expression and play crucial roles in cancer development by serving as miRNA sponges that sequester miRNA molecules, and thereby affect the stability of target mRNAs and dynamically regulate mRNA translation.^9^, ^10^ The most investigated circRNAs include CDR1as, circ‐Foxo3, cir‐ITCH and circHIPK3, which are associated with hepatocellular carcinoma, lung cancer and osteosarcoma via their interactions with miR‐7, FOXO3, miR‐17 and miR‐124 respectively.^11^ Several other circRNAs have been reported to be associated with gastric cancer development. For example, circPVRL3 expression was found to be correlated with GC incidence, a higher TNM stage and lower overall survival rates.^12^ The levels of hsa_circ_000745 expression in GC tissues were found to be upregulated and correlate with tumour differentiation, while the levels of hsa_circ_000745 expression in plasma correlated with the stage of tumour‐node‐metastasis.^13^ Recently, circRNA_100269 was found to be downregulated in gastric cancer and suppresses tumour cell growth by targeting miR‐630.^14^ However, the roles played by circRNAs in GC progression require further investigation.

In this study, we screened the circRNAs‐miRNA network that is reported to exist during the transformation of human gastric epithelial cells and found that circRNA hsa_circ_006100 was significantly upregulated in cancerous cells, while miR‐195 expression was reduced. We subsequently investigated the roles of hsa_circ_006100 and miR‐195 in gastric cancer tissues and corresponding adjacent non‐cancerous tissues (n = 20). In addition, hsa_circ_006100 knockdown and overexpression studies were performed to determine the function of _hsa_circ_006100 in GC cell lines in vitro and in vivo. We found that circRNA hsa_circ_006100 functions as an oncogene to promote GC cell proliferation and metastasis via regulation of miR‐195 and its target gene *GPRC5A*.

## 2 MATERIALS AND METHODS

### 2.1 Cell culture and tissue collection

Four GC cell lines (KN‐45, MGC‐803, AGS and SGC‐7901) and one human gastric epithelial cell line (GES‐1‐T) were used in this study. The cells were cultured in Dulbecco's modified Eagle's medium (DMEM; HyClone, Thermo Fisher Scientific) containing 10% foetal bovine serum (FBS; Gibco‐Invitrogen Corp.) at 37°C in an atmosphere of 5% CO_2_. Twenty pairs of GC tissue and adjacent non‐tumour tissue were collected from patients who underwent surgery from September 2015 to May 2018 in the Fifth Affiliated Hospital of Guangzhou Medical University and Guangdong Provincial People's Hospital. The protocol for this study was approved by the Biomedical Ethics Committee of the Fifth Affiliated Hospital of Guangzhou Medical University, and a signed informed consent document was obtained from each patient. No patient had received chemotherapy or radiotherapy prior to surgery.

### 2.2 Reagents

The binding sites for miR‐195 in the Renilla luciferase reporter vectors psiCHECK2‐GPRC5A (mutant) and psiCHECK2‐hsa_circ_006100‐WT (wild type) were constructed by GenePharma. Oligonucleotide sequences of the miR‐195 mimics, inhibitors and NC (negative control) were purchased from GenePharma. The lentivirus vectors used for transfection of siRNA‐hsa_circ_006100, miR‐195 mimics, the inhibitor or negative control were constructed by GeneChem. Antibodies against PCNA, caspase‐3, BCL‐2, E‐cadherin and N‐cadherin were obtained from Cell Signaling Tech and Abcam.

### 2.3 The bioinformatics procedures and algorithms

The EBseq algorithm was applied to filter the differentially expressed genes, and gene co‐expression networks were used to assess the relationships among different miRNAs and ncRNAs according to the normalized expression values of genes selected from genes associated with significant GO terms and pathways. Core regulatory factors were determined by the degree of differences between two class samples.

### 2.4 RNA isolation and qRT‐PCR

Total RNA was extracted using Trizol reagent (Invitrogen) according to a standard RNA isolation protocol. The synthesis of cDNA from 2 μg of total RNA was performed using a High‐Capacity RNA‐to‐cDNA™ Kit (Thermo Fisher Scientific) according to the manufacturer's instructions. The relative levels of mRNA expression were determined by the qRT‐PCR performed using TaqMan™ Fast Advanced Master Mix (Thermo Fisher Scientific). The levels of hsa_circ_006100, miR‐195 and GPRC5A expression were normalized to those of GAPDH and U6. The primers used for qRT‐PCR are listed in Table 1 **.**

**Table 1:** Sequences of primers used for quantitative RT‐PCR assay

Primer ID	Primer sequences (5′‐3′)
Circ‐006100 F	ATAACAACTGCTCAGAGTGCGA
Circ‐006100 R	CTCAGCTTCCTGTAGGATGGTC
miR‐195 F	ACACTCCAGCTGGGTAGCAGCACAGAAATAT
miR‐195 R	CTCAACTGGTGTCGTGGA
U6 F	CTCGCTTCGGCAGCACA
U6 R	AACGCTTCACGAATTTGCGT
GPRC5A F	CTCACTCTCCCGATCCTCGT
GPRC5A R	CAGTCCGATGATGAAGGCGAA
GAPDH F	TGTTCGTCATGGGTGTGAAC
GAPDH R	ATGGCATGGACTGTGGTCAT

### 2.5 CCK‐8 assay

MGC‐803 and AGS cells were seeded into the wells of a 96‐well plate (2 x 10^3^ cells/well) and cultured to ~80% confluence. The cells were then transfected with pcDNA3.1‐hsa_circ_006100, miR‐195 mimics, the inhibitor or the negative control at a concentration of 1 ng/mL for the specified time period by using Lipofectamine 2000 (Invitrogen) according to the manufacturer's instructions. After the indicated incubation time, CCK‐8 solution (10 μL) was added to each well and the cultures were incubated at 37°C for 90 minutes. The OD value of each well at 450 nm was detected with a microplate reader.

### 2.6 Colony formation assay

MGC‐803 and AGS cells were harvested, re‐suspended in complete medium containing 10% FBS and subsequently seeded into 12‐well plates (50‐100 cells per well). The cells were then cultured under standard conditions for 10 days; after which, they were stained with 0.1% crystal violet, fixed in methanol for 15 minutes at room temperature and then visualized under a dissection microscope (Olympus). Cell colonies consisting of >50 cells were counted and used for comparisons of colony formation ability.

### 2.7 Luciferase reporter assay

MGC‐803 and AGS cells were co‐transfected with vectors containing the negative control construct, pcDNA3.1‐hsa_circ_006100, psiCHECK2‐GPRC5A and miR‐195 mimics. Luciferase activity was measured using a Dual‐Luciferase Reporter Assay System (Promega) according to the manufacturer's instructions. Briefly, firefly luciferase acted as a reporter gene for the normalized control.

### 2.8 Hoechst staining assay

MGC‐803 and AGS cells transfected with the specified vectors were cultured at 37°C for 48 hours and then stained with 0.1 μg/mL Hoechst 33342 (Sigma). The apoptosis rates of MGC‐803 and AGS cells were evaluated under a fluorescence microscope (OLYMPUS IX71; Olympus Corporation) equipped with a filter for Hoechst 33342 (365 nm). Changes that occurred in nuclear morphology were used for assessing the apoptosis rates of MGC‐803 and AGS cells.

### 2.9 Transwell assay

The migration and invasion capabilities of MGC‐803 and AGS cells were analysed using a Transwell culture system. For assessing cell migration, 3 × 10^4^ MGC‐803 or AGS cells that had been cultured in serum‐free medium were added to the upper chamber, and culture medium supplemented with 10% FBS was added to the lower chamber. After culture, cells in the lower chamber were stained by crystal violet and observed under an inverted microscope. For analysis of invasion capability, the inner sides of the Transwell chamber were first coated with Matrigel Basement Membrane Matrix (BD Biocoat). Next, cells were then placed in the upper chamber, and the lower chamber was filled with culture medium. After a specified culture time, the number of cells in the lower chamber was counted under an inverted microscope.

### 2.10 Western blot studies

To determine the expression of BCL‐2, caspase‐3 and EMT markers, total proteins were isolated from tissues using RIPA buffer. A sample of total proteins (~30 µg) extracted from each tissue specimen was boiled at 100°C for 5 minutes and then separated by SDS‐PAGE (SDS‐polyacrylamide gel electrophoresis) on a 12% polyacrylamide gel. The separated protein bands were transferred onto a PVDF membrane (Millipore) that was subsequently blocked with 5% lipid‐free milk solution. The membrane was then incubated overnight with primary antibodies at 4°C. After incubation, the membrane was washed three times with TBST buffer and then incubated with a HRP‐conjugated secondary antibody for 2 hours at room temperature. The membrane was then washed three more times with TBST buffer, and the protein blots were visualized using ECL‐Plus reagent (Millipore). GAPDH was used as a loading control in all the Western blot studies. The antibodies used for the Western blot studies were as follows: anti‐Bcl‐2 (#ab32124; Abcam), anti‐caspase‐3 (#ab13847; Abcam), anti‐GPRC5A (#orb84936; Biorbyt), anti‐EGFR (#ab52894; Abcam), anti‐E‐cadherin (#ab1416; Abcam), anti‐N‐cadherin (#ab18203; Abcam) and anti‐GAPDH (#ab9485; Abcam).

### 2.11 Immunohistochemistry

PCNA expression in tumour tissues was analysed via IHC staining performed as previously described.^15^ Tissue specimens were fixed in formalin, embedded with paraffin, cut into 2‐μm‐thick sections and then mounted onto slides. The slides with mounted tissue specimens were incubated in xylene for 5 minutes, washed twice in 100% ethanol for 10 minutes and then washed in 95% ethanol for 10 minutes. Antigen unmasking was performed, and the slides were then blocked with 3% hydrogen peroxide for 30 minutes at room temperature. Subsequently, the FFPE sections were incubated with the primary antibody against PCNA at 4℃ overnight. Finally, the tissue sections were stained with DAB chromogen contained in an EnVision Detection System kit (DAKO), and the cell nucleus was stained with haematoxylin.

### 2.12 Tumour model

Female athymic nude mice (3 weeks old) purchased from the Experimental Animal Center of the Southern Medical University were used to establish the tumour model. MGC‐803 cells were infected with lentivirus‐RNAi‐siRNA‐hsa_circ_006100, miR‐195 mimics, the inhibitor or negative control (MOI = 100). Next, 2 × 10^6^ MGC‐803 cells were subcutaneously injected into the rear flank of each nude mouse (6 mice per group). The mice were sacrificed four weeks later, at which time, their tumour volumes were analysed, tissue specimens were collected for use in IHC assays, and samples of total protein were obtained for use in Western blot studies. Results for tumour size are presented as the mean tumour size (mm^3^) calculated as (V [cm^3^] = width^2^ [cm^2^] × length [cm]/ 2)for each group at various time points until the end of the experiment.

### 2.13 Statistical analysis

All results were analysed using SPSS for Windows, version 16.0 (SPSS Inc) and Prism, version 5.0 software (GraphPad Software Inc). The unpaired *t* test or the Mann‐Whitney *U* test was used for comparisons between two groups, and one‐way ANOVA was used for multiple group comparisons. A *P*‐value <0.05 was considered statistically significant. All experiments were repeated at least three times.

## 3 RESULTS

### 3.1 Hsa_circ_006100 was upregulated and miR‐195 was downregulated in malignantly transformed gastric cells

**Figure 1:**
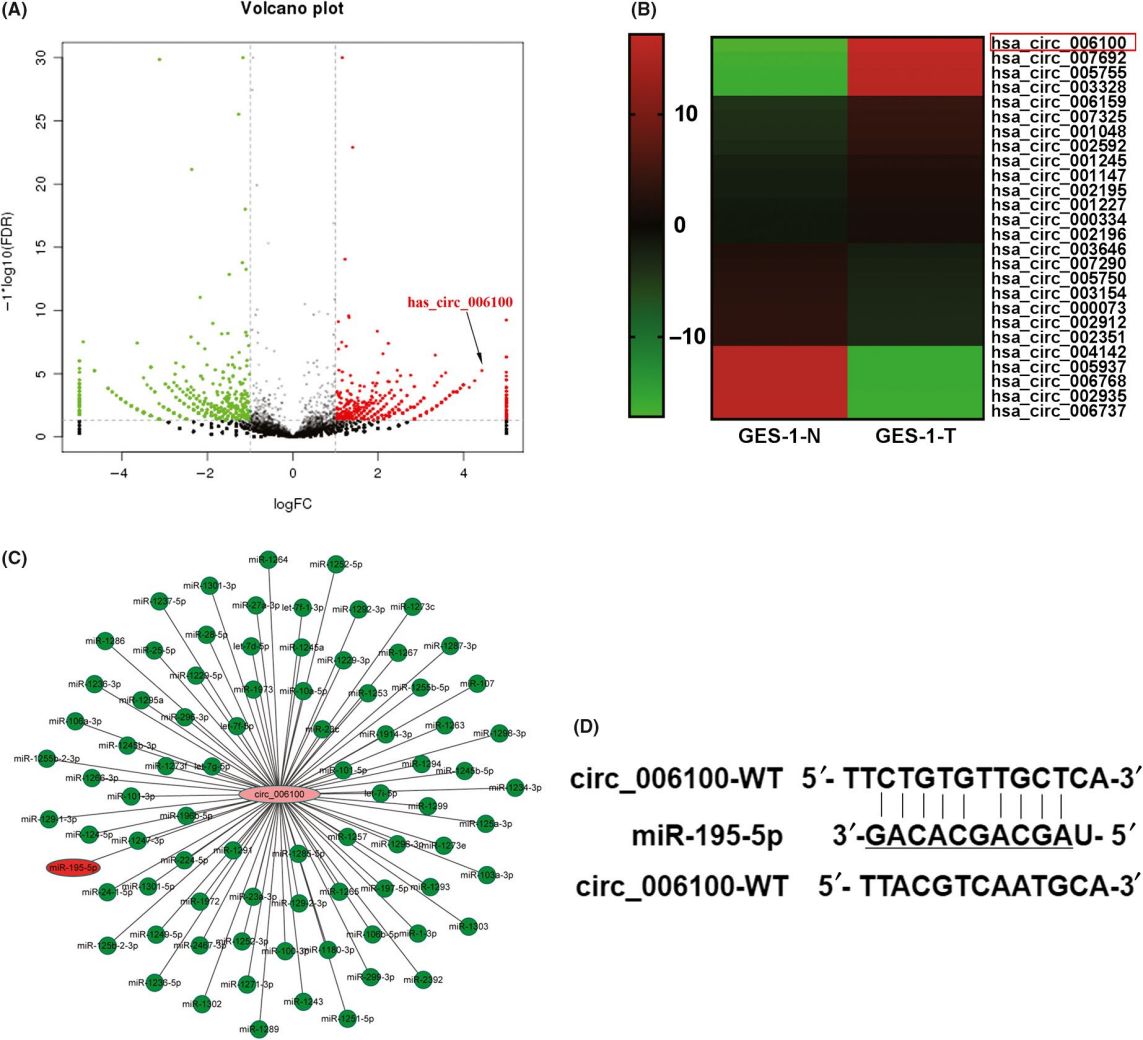
Hsa_circ_006100 was upregulated during the development of gastric cancer. CircRNAs that were differentially expressed in GES‐1‐T cells vs GES‐1‐N cells were characterized by high‐throughput RNA sequencing and defined by showing a ≥2‐fold change or <0.5‐fold change in expression, with a *P*‐value <0.05 and a FDR value <6.42^−7^. Three samples from each group had their RNA sequenced. A,B, After high‐throughput RNA sequencing, a volcano map was created and analysed according to the expression of circRNAs and their fold changes. The levels of circRNA hsa_circ_006100 were enhanced in malignantly transformed human gastric epithelial cells (GES‐1‐T). C,D, The circRNA‐miRNA co‐expression network for differentially expressed circRNAs and miRNAs in GES‐1‐T cells. Reduced miR‐195 levels were related to an upregulation of hsa_circ_006100, which has potential target sites

We previously investigated the role of ncRNAs in malignantly transformed human gastric epithelial cells (GES‐1‐T cells) and used high‐throughput RNA sequencing to examine differences in ncRNA expression between GES‐1‐T cells and GES‐1‐N cells. Here, we focused on differentially expressed circRNAs that displayed a ≥2‐fold or <0.5‐fold change in their expression level, with a *P*‐value <0.05. A volcano map was constructed showing the expression and fold change of each circRNA. The map showed that hsa_circ_006100 (1043 bp in length, locus: 12887588 ~ 12888630 at Chromosome 12) was significantly upregulated circRNA in malignantly transformed GES‐1‐T cells (Figure 1A,B). Simultaneously, a circRNA‐miRNA co‐expression network was created and analysed. It showed that cancer‐related miRNA‐195 was negatively co‐expressed with hsa_circ_006100 (Figure 1C). A bioinformatics analysis of possible binding targets indicated that hsa_circ_006100 harbours a target site for miR‐195, suggesting that hsa_circ_006100 might interact with miR‐195 during the malignant transformation of human gastric epithelial cells (Figure 1D).

### 3.2 High hsa_circ_006100 and low miR‐195 expression predict a poor clinical outcome

**Figure 2:**
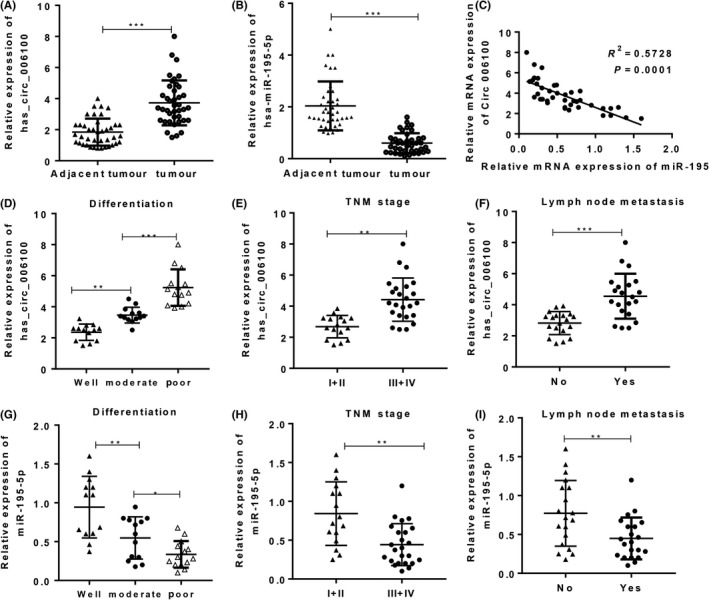
High hsa_circ_006100 levels and low miR‐195 levels predicted a poor clinical outcome. Samples of total RNA from gastric cancer (GC) tissues and corresponding non‐cancerous tissues were used for cDNA synthesis. The levels of hsa_circ_006100 (A) and miR‐195 (B) in GC tissues were determined by quantitative RT‐PCR (n = 20). C, The correlation between hsa_circ_006100 and miR‐195 expression in the GC tissues and corresponding non‐cancerous tissues was analysed (n = 20). The correlations between hsa_circ_006100 (D‐F) or miR‐195 (G‐I) and differentiation, TNM and LNM were analysed. Data are expressed as the mean ± SD** *P* < 0.01, *** *P* < 0.001

To investigate the clinical role of hsa_circ_006100/miR‐195 during the development of gastric cancer (GC), samples of gastric cancer and corresponding non‐cancerous tissues (n = 20) were collected and analysed for their expression of hsa_circ_006100 and miR‐195. Clinical data pertaining to those specimens, including the patient's gender, age, tumour size, distant metastasis and other information, are listed in Tables 2 and 3. Our results showed that GC tissues had higher levels of hsa_circ_006100 expression and lower levels of miR‐195 expression when compared with the non‐cancerous tissues (Figure 2A,B). An analysis of the correlation between hsa_circ_006100 and miR‐195 showed that hsa_circ_006100 was negatively correlated with miR‐195 expression in the GC tissues (Figure 2C). In addition, patients with high levels of hsa_circ_006100 expression or low levels of expression of miR‐195 expression had worse clinical outcomes, were at an advanced TNM stage, showed poor cell differentiation and had lymph node metastases (Figure 2 D‐H). These findings indicated that hsa_circ_006100 participates in the progression of GC, while miR‐195 might function as a tumour suppressor in GC.

**Table 2:** Correlation of the expression of circ_006100 with clinicopathologic features

Clinic‐pathological parameters	High circ_006100 expression	Low circ_006100 expression	*P*‐value
Gender			
Female	10	12	0.3239
Male	11	7	
Age (y)			
≥57	10	5	0.1646
<57	11	14	
Tumour size (cm^3^)			
≥3	16	5	*0.0016
<3	5	14	
Differentiation			
Well	0	13	***<0.0001
Moderate	7	6	
Poor	14	0	
TNM			
I + II	2	14	***<0.0001
III + IV	19	5	
LNM			
No	4	15	**<0.0002
Yes	17	4	

* *P* < 0.01.

** *P* < 0.001.

*** *P* < 0.0001.

**Table 3:** Correlation of the expression of miR‐195‐5p with clinicopathologic features

Clinic‐pathological parameters	High miR‐195‐5p expression	Low miR‐195‐5p expression	*P*‐value
Gender			
Female	10	10	>0.9999
Male	10	10	
Age (y)			
≥57	9	11	>0.5271
<57	11	9	
Tumour size (cm^3^)			
≥3	8	13	0.1134
<3	12	7	
Differentiation			
Well	11	2	**0.0011
Moderate	8	6	
Poor	2	12	
TNM			
I + II	12	4	**0.0098
III + IV	8	16	
LNM			
No	13	6	*0.0267
Yes	7	14	

* *P* < 0.05.

** *P* < 0.01.

### 3.3 Hsa_circ_006100 inhibited the tumour suppressive function of miR‐195 in gastric cancer cells

**Figure 3:**
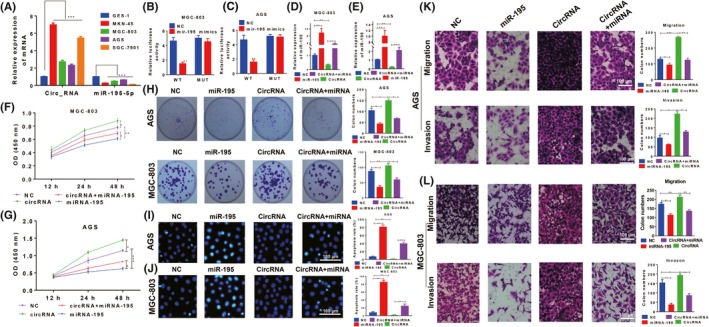
Hsa_circ_006100 promoted cell growth and metastasis via miR‐195. A, The relative levels of hsa_circ_006100 in four GC cell lines (MKN‐45, MGC‐803, AGS and SGC‐7901) and GES‐1‐T human gastric epithelial cells were determined by quantitative RT‐PCR. B,C, MGC‐803 (B) and AGS cells (C) were transfected with miR‐195 mimics and full‐length (wild‐type or mutant) psiCHECK2‐hsa_circ_006100. The interaction between miR‐195 mimics and hsa_circ_006100 was determined by the luciferase reporter assay. D,E, Suppression of miR‐195 expression by hsa_circ_006100 overexpression in MGC‐803 (D) and AGS cells (E). MGC 803 and AGS cells were transfected with hsa_circ_006100 and/or miR‐195 mimics, and the levels of miR‐195 in the cells were determined by quantitative RT‐PCR. E,F, The viability of MGC‐803 (F) and AGS cells (G) transfected with hsa_circ_006100 and/or miR‐195 mimics was determined with the CCK‐8 assay (H) The colony formation ability of MGC‐803 and AGS cells transfected with hsa_circ_006100 and/or miR‐195 mimics. I,J, The apoptosis of MGC‐803 (I) and AGS cells (J) transfected with hsa_circ_006100 and/or miR‐195 mimics as determined by the Hoechst assay. K,L, The migration and invasion capabilities of MGC‐803 (K) and AGS cells (L) as determined by Transwell assays. Data are expressed as the mean ± SD NC, negative control; MUT, mutant; WT, wild type; *P < 0.05, **P < 0.01, ***P < 0.001

We next used GES‐1‐T cells and four GC cell lines (MKN‐45, MGC‐803, AGS and SGC‐7901) to determine the functions of hsa_circ_006100 and miR‐195 in vitro. An upregulated hsa_circ_006100 level and downregulated miR‐195 level were found in all four GC cell lines (Figure 3A). The MGC‐803 and AGS cell lines displayed high levels of hsa_circ_006100 expression were chosen for further study. Luciferase reporter assays showed that miR‐195 mimics significantly impaired the luciferase activity of psiCHECK2‐hsa_circ_006100 with wild‐type miR‐195 binding sites, and this was not observed for psiCHECK2‐hsa_circ_006100 with mutant type binding sites. These results indicated that miR‐195 could interact with hsa_circ_006100 (Figure 3B,C). Overexpression of hsa_circ_006100 in these two cell lines decreased the miR‐195 levels (Figure 3D,E). The viability (Figure 3 F,G) and proliferation rates (Figure 3H) of MGC‐803 and AGS cells were also enhanced by hsa_circ_006100, but were suppressed by miR‐195. miR‐195 induced apoptosis in the two cell lines, but cell proliferation was restored by overexpression of hsa_circ_006100 (Figure 3 I,J). We also analysed cell migration and invasion capabilities (Figure 3 K,L). Results of Transwell assays showed that upregulation of hsa_circ_006100 in the two cell lines increased their migration and invasion abilities, while these abilities were impaired by miR‐195 mimics. These in vitro results suggested that hsa_circ_006100 promotes GC cell growth and metastasis by inhibiting miR‐195.

### 3.4 Oncogene GPRC5A was a target gene of miR‐195

**Figure 4:**
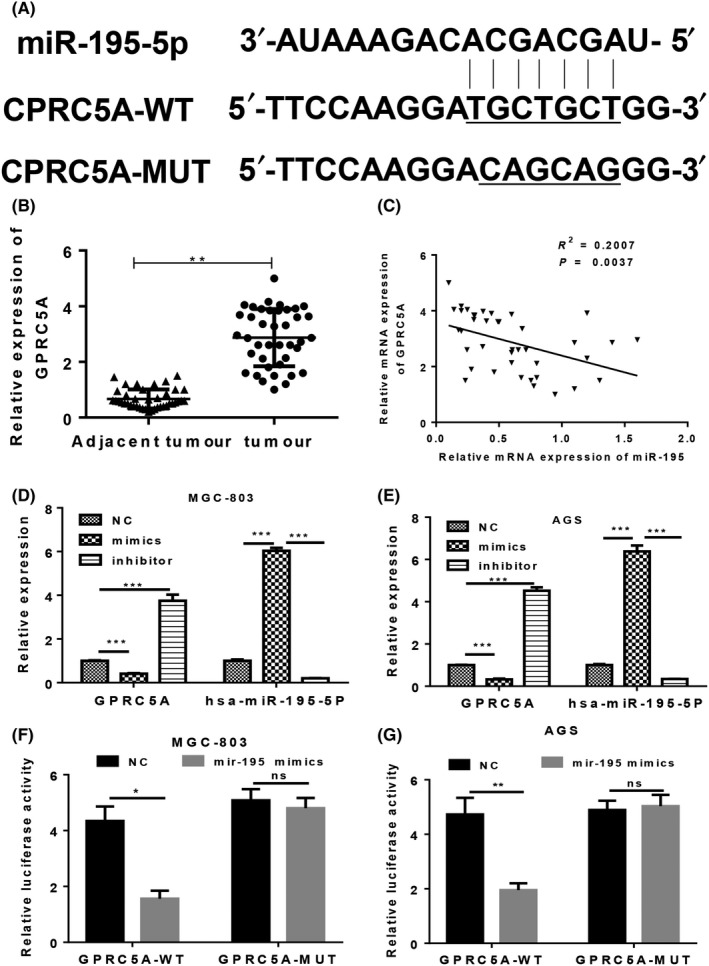
GPRC5A was targeted by miR‐195 in GC cells. A, The interaction of miR‐195 with GPRC5A gene sequences as predicted by bioinformatics. B, The GPRC5A mRNA levels in GC tissues and adjacent tissues were determined by quantitative RT‐PCR (n = 40). C, The correlation between GPRC5A and miR‐195 expression levels in GC and adjacent tissues. D,E, Overexpression of miR‐195 in MGC‐803 and AGS cells decreased the expression of GPRC5A. MGC‐803 (D) and AGS cells (E) were transfected with miR‐195 mimics and the inhibitor, respectively. F,G, MGC‐803 (F) and AGS cells (G) were transfected with miR‐195 mimics and the wild‐type or mutant full‐length 3’‐UTR of psiCHECK2‐GPRC5A; after which, luciferase reporter assays were performed to confirm the direct target sites. Data are expressed as the mean ± SD GPRC5A, G protein‐coupled receptor, family C, group 5, member A; MUT, mutant; NC, negative control; WT, wild type. *P < 0.05, **P < 0.01, ***P < 0.001

Previous studies indicated that the G protein‐coupled receptor, class C, group 5, member A (GPRC5A) plays important pathogenic roles, and its dysregulation can promote the development of several different types of cancer via its effect on EGFR signalling.^16^ Our bioinformatics study showed that *GPRC5A* was a target gene of miR‐195 in GC cells; we also found that GPRC5A expression was upregulated in GC tissues (Figure 4A,B). Furthermore, miR‐195 levels were negatively associated with GPRC5A expression (Figure 4C). Transfection with miR‐195 mimics inhibited the expression of GPRC5A in MGC‐803 and AGS cells, while transfection with the miR‐195 inhibitor promoted GPRC5A expression in the two cell lines (Figure 4D,E). A luciferase reporter vector of GPRC5A with full‐length wild‐type or mutant miR‐195 binding sites was co‐transfected with miR‐195 mimics into MGC‐803 and AGS cells (Figure 4 F,G). The subsequent results showed that miR‐195 mimics could significantly impair the luciferase activity of the wild‐type reporter, but not of the mutant type reporter, indicating that *GPRC5A* is a target gene of miR‐195 in GC cells.

### 3.5 GPRC5A contributes to the tumour suppressor role of miR‐195 in gastric cancer

**Figure 5:**
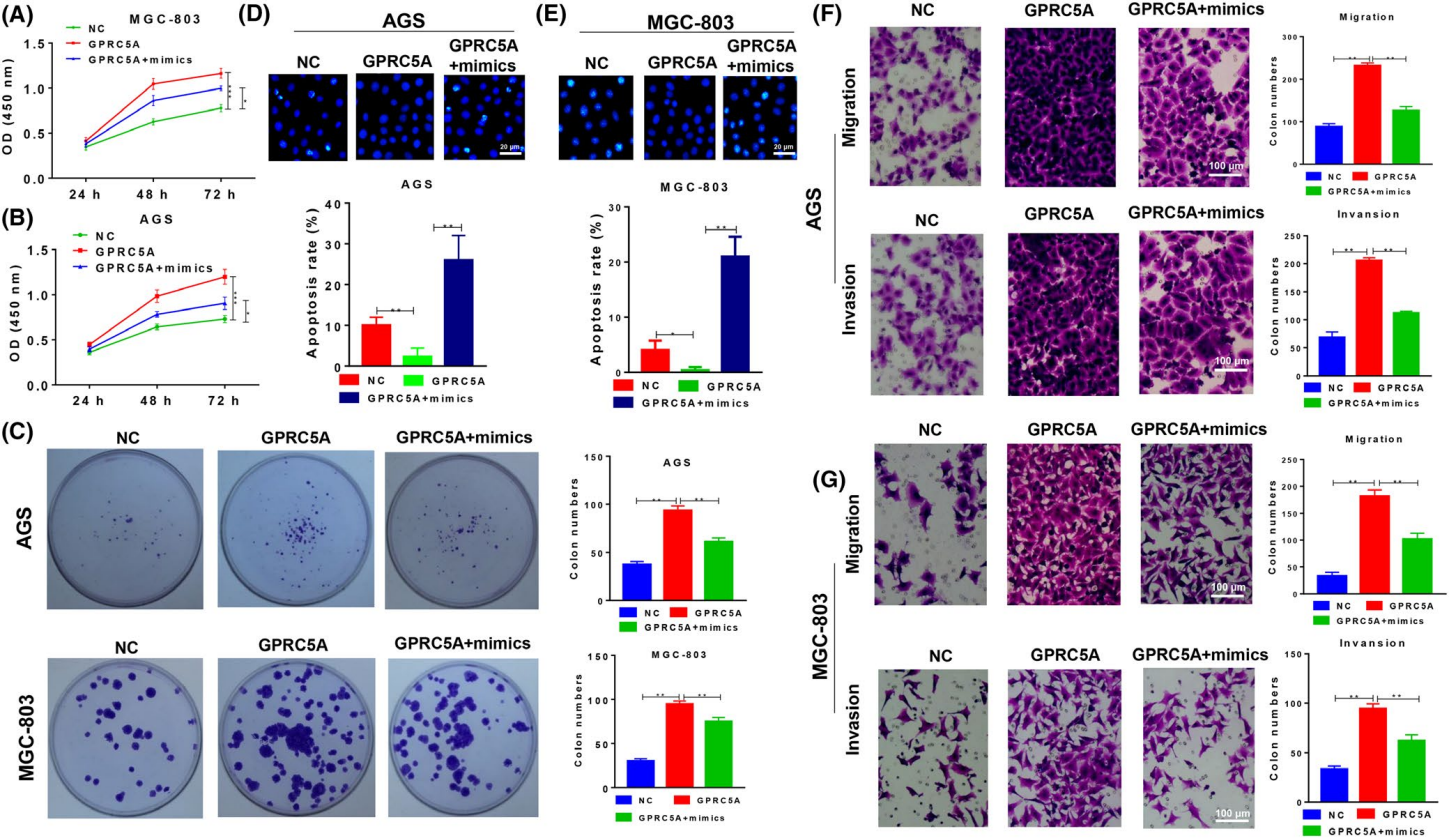
The tumour suppressor miR‐195 inhibited the function of GPRC5A. A,B, The viability of MGC‐803 cells (A) and AGS cells (B) transfected with GPRC5A and/or miR‐195 mimics as determined by the CCK‐8 assay. C, The colony formation ability of MGC‐803 and AGS cells transfected with GPRC5A and/or miR‐195 mimics as determined by the Hoechst assay. D,E, Apoptosis of AGS cells (D) and MGC‐803 cells (E) transfected with GPRC5A and/or miR‐195 mimics as determined by the Hoechst assay. F,G, The migration and invasion abilities of AGS (F) and MGC‐803 cells (G) transfected with GPRC5A and/or miR‐195 mimics as determined by the Transwell assay. GPRC5A, G protein‐coupled receptor, family C, group 5, member A; NC, negative control; data are expressed as the mean ± SD*P < 0.05, **P < 0.01, ***P < 0.001

The function of miR‐195/GPRC5A signalling in GC cells was analysed. The viability (Figure 5A,B) and proliferation rates (Figure 5C) of MGC‐803 and AGS cells were enhanced when GPRC5A was overexpressed, but this effect was repressed by the miR‐195 mimics. Apoptosis showed similar trends in both two cell lines (Figure 5D,E). The migration and invasion abilities of MGC‐803 and AGS cells were also increased by the overexpression of GPRC5A, but were impaired by the `miR‐195 mimics (Figure 5 F,G). These in vitro results suggested that miR‐195 suppresses GC cell growth via its effect on GPRC5A.

### 3.6 Knockdown of hsa_circ_006100 inhibited tumour growth in vivo via miR‐195 signalling

**Figure 6:**
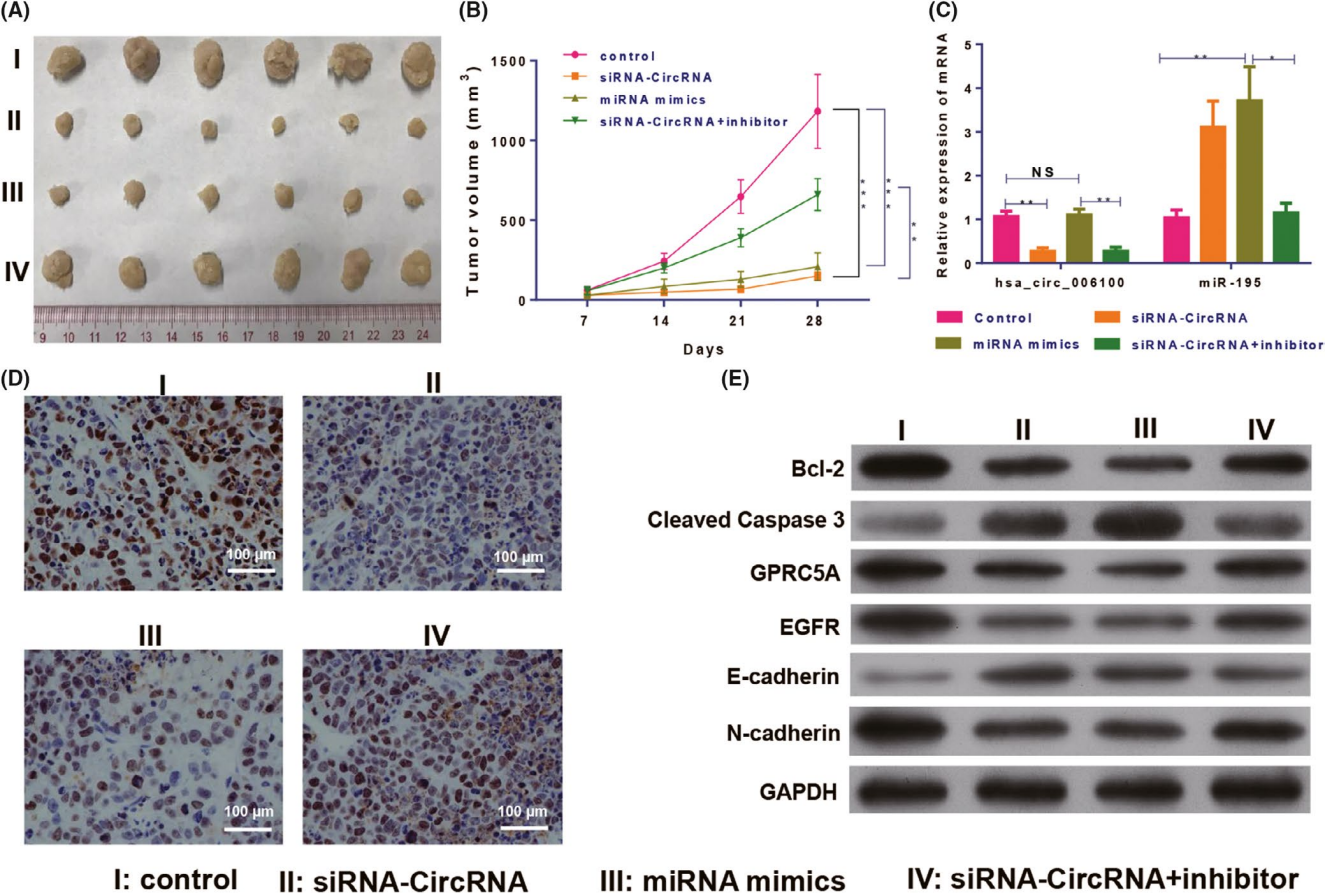
Knockdown of hsa_circ_006100 inhibited gastric cancer growth via miR‐195/GPRC5A signalling. A, Tumours derived from MGC‐803 cells treated with lentivirus‐siRNA‐hsa_circ_006100 and/or miR‐195 inhibitor/mimics in the xenograft model (B) Volumes of tumours derived from MGC‐803 cells treated with lentivirus‐siRNA‐hsa_circ_006100 and/or miR‐195 inhibitor/mimics. C, Expression of circRNA hsa_circ_006100 and miR‐195 in tumours derived from MGC‐803 cells treated with lentivirus‐siRNAhsa_ circ_006100 and/or miR‐195 inhibitor/mimics. D, Expression of the proliferation indicator PCNA in tumours derived from MGC‐803 cells treated with lentivirus‐siRNA‐hsa_circ_006100 and/or miR‐195 inhibitor/mimics, as determined by IHC assays. E, Levels of the anti‐apoptosis proteins BCL‐2 and caspase‐ 3, as well as EGFR and EMT proteins, in tumours derived from MGC‐803 cells treated with lentivirus‐siRNAhsa_ circ_006100 and/or miR‐195 inhibitor/mimics, as determined by Western blotting. GAPDH was used as the internal standard. Mice harbouring tumours were sacrificed four weeks after model establishment. Bcl‐2, B‐cell lymphoma‐2; EGFR, epidermal growth factor receptor; GAPDH, glyceraldehyde‐3‐phosphate dehydrogenase; GPRC5A, G protein‐coupled receptor, family C, group 5, member A; NC, negative control; data are expressed as the mean ± SD * *P* < 0.05, ** *P* < 0.01

The tumorigenic function of hsa_circ_006100 in GC was analysed in vivo. MGC‐803 cells transfected with lentivirus‐siRNA‐hsa_circ_006100 and/or miR‐195 inhibitor/mimics were injected into athymic nude mice to establish the xenograft model. Measurements of tumour growth showed that knockdown of hsa_circ_006100 and overexpression of miR‐195 significantly restricted the tumour volumes and inhibited tumour growth. However, inhibition of miR‐195 restored the tumorigenic activity of hsa_circ_006100 (Figure 6A,B). The expression of a proliferation indicator (PCNA, Figure 6C) and an anti‐apoptosis protein (BCL‐2) was analysed in each group of tumour tissues. Hsa_circ_006100 was found to positively regulate PCNA and BCL‐2 levels in the tumour tissues via miR‐195. GPRC5A expression and downstream EGFR expression were inactivated by hsa_circ_006100 knockdown, but were restored by miR‐195 mimics, inducing expression of the EMT phenotype in GC cells in vivo (Figure 6D).

## 4 DISCUSSION

Gastric cancer (GC) accounts for ~7% of all new cancer cases and contributes to 9% of all deaths due to cancer. Even with the rapid development of new technologies and our deeper understanding of molecular biology, the exact mechanisms of GC development remain unclear, which greatly limits our ability to treat GC.^1^ Non‐coding RNAs become dysregulated during all processes involved in carcinogenesis and thus represent promising biomarkers for use in the diagnosis and treatment of cancer.^17^ In this study, hsa_circ_006100 was identified as an oncogene that promotes GC cell proliferation and metastasis via regulation of miR‐195/GPRC5A signalling.

We previously found that the during the malignant transformation of human gastric epithelial cells, LncRNA LOC101927497 functions as a suppressor by interacting with miR‐574 to inhibit the proliferation and migration of GC cells, which suggests a regulatory function for ncRNAs in GC progression.^8^ Similarly, circRNAs also exhibit aberrant expression in multiple types of cancer, including colorectal cancer, hepatocellular carcinoma, gastric cancer and pancreatic ductal adenocarcinoma.^18^ The levels of circ_0067934 expression in oesophageal squamous cell carcinoma (ESCC) tissues are significantly higher than those in normal tissues and are correlated with ESCC differentiation, T stage and TNM stage.^19^ Xu et al^20^ reported that upregulated ciRS‐7 expression in hepatocellular carcinoma (HCC) tissues with concurrent hepatic microvascular invasion (MVI) was inversely correlated with miR‐7 expression.^20^ The levels of circ_002059 expression in the plasma of postoperative gastric cancer patients were found to be significantly higher than those in the plasma of preoperative gastric cancer patients and were also associated with distant metastasis, TNM stage, patient gender and age.^21^ In this study, we found that hsa_circ_006100 expression was enhanced during the development of GC, and these enhanced levels were predictive of a poor clinical outcome for GC patients. Overexpression of hsa_circ_006100 increased the viability, colony formation ability and migration/invasion ability of two GC cell lines.

During the past decade, several studies have reported interactions that occur among mammalian lncRNAs/circRNAs and miRNAs. LncRNAs/circRNAs are targeted by miRNAs to reduce their stability and also function as molecular decoys or sponges of miRNAs.^22^ CircRNA Cdr1as is massively bound by miR‐7 and miR‐671 in human and mouse brain tissues.^23^ In osteoarthritis (OA), circRNA‐CER was reported to function as a sponge by competitively binding miR‐136 and could also target and regulate MMP13 via TGFβ, JNK and ERK pathways.^24^ Here, we found that miR‐195 levels were reduced in GC tissues when compared with their levels in adjacent non‐cancerous tissues and that miR‐195 could target hsa_circ_006100 in GC cells. Overexpression of hsa_circ_006100 reduced the miR‐195 levels in two GC cell lines. GPRC5A in samples of non‐small cell lung carcinoma (NSCLC) tissue was expressed at lower levels than in corresponding adjacent normal tissues and functioned as a tumour suppressor. Knockdown of GPRC5A expression in mice leads to the activation of NF‐κB signalling and promotes lung inflammation and tumorigenesis.^25^ In colorectal cancer, GPRC5A functions as an oncogene and is abundantly present in tumour epithelium, with the neuroendocrine cells showing strong staining of GPRC5A on the plasma membrane.^26^ Cheng et al^16^ reported that GPRC5A functions as a tumour suppressor in GC. In our study, miR‐195 was found to target GPRC5A and thereby inhibit GC cell proliferation and metastasis. Knockdown of hsa_circ_006100 in vivo inhibited tumour growth by upregulating expression of the tumour suppressor miR‐195; this led to reduced GPRC5A levels and a downregulation the EMT phenotype.

## 5 CONCLUSIONS

In summary, we found that hsa_circ_006100 induces the malignant transformation of gastric cells and exerts this effect by inhibiting expression of the tumour suppressor, miR‐195. Inhibition of miR‐195 expression induced GPRC5A to increase cell viability and promote the proliferation and metastasis of GC cells.

## CONFLICT OF INTEREST

No competing financial interests exist.

## References

[1] Karimi P , Islami F , Anandasabapathy S , Freedman ND , Kamangar F . Gastric cancer: descriptive epidemiology, risk factors, screening, and prevention. Cancer Epidemiol Biomarkers Prev. 2014;23(5):700‐713. 24618998 10.1158/1055-9965.EPI-13-1057 PMC4019373

[2] Pasechnikov V , Chukov S , Fedorov E , Kikuste I , Leja M . Gastric cancer: prevention, screening and early diagnosis. World J Gastroenterol. 2014;20(38):13842‐13862. 25320521 10.3748/wjg.v20.i38.13842 PMC4194567

[3] Hamashima C . Current issues and future perspectives of gastric cancer screening. World J Gastroenterol. 2014;20(38):13767‐13774. 25320514 10.3748/wjg.v20.i38.13767 PMC4194560

[4] Bertuccio P , Chatenoud L , Levi F , et al. Recent patterns in gastric cancer: a global overview. Int J Cancer. 2009;125(3):666‐673. 19382179 10.1002/ijc.24290

[5] Wheeler DA , Wang L . From human genome to cancer genome: the first decade. Genome Res. 2013;23(7):1054‐1062. 23817046 10.1101/gr.157602.113 PMC3698498

[6] Ding L , Ren J , Zhang D , et al. A novel stromal lncRNA signature reprograms fibroblasts to promote the growth of oral squamous cell carcinoma via LncRNA‐CAF/interleukin‐33. Carcinogenesis. 2018;39(3):397‐406. 29346528 10.1093/carcin/bgy006

[7] Bartonicek N , Maag JL , Dinger ME . Long noncoding RNAs in cancer: mechanisms of action and technological advancements. Mol Cancer. 2016;15(1):43. 27233618 10.1186/s12943-016-0530-6 PMC4884374

[8] Luo Y , Liang M , Yao W , et al. Functional role of lncRNA LOC101927497 in N‐methyl‐N'‐nitro‐N‐nitrosoguanidine‐induced malignantly transformed human gastric epithelial cells. Life Sci. 2018;193:93‐103. 29223541 10.1016/j.lfs.2017.12.007

[9] Yang Z , Xie L , Han L , et al. Circular RNAs: Regulators of Cancer‐Related Signaling Pathways and Potential Diagnostic Biomarkers for Human Cancers. Theranostics. 2017;7(12):3106‐3117. 28839467 10.7150/thno.19016 PMC5566109

[10] Zhang Y , Liang W , Zhang P , et al. Circular RNAs: emerging cancer biomarkers and targets. J Exp Clin Cancer Res. 2017;36(1):152. 29096676 10.1186/s13046-017-0624-z PMC5667461

[11] Wang Y , Mo Y , Gong Z , et al. Circular RNAs in human cancer. Mol Cancer. 2017;16(1):25. 28143578 10.1186/s12943-017-0598-7 PMC5282898

[12] Sun HD , Xu ZP , Sun ZQ , et al. Down‐regulation of circPVRL3 promotes the proliferation and migration of gastric cancer cells. Sci Rep. 2018;8(1):10111. 29973643 10.1038/s41598-018-27837-9 PMC6031698

[13] Huang M , He YR , Liang LC , Huang Q , Zhu ZQ . Circular RNA hsa_circ_0000745 may serve as a diagnostic marker for gastric cancer. World J Gastroenterol. 2017;23(34):6330‐6338. 28974900 10.3748/wjg.v23.i34.6330 PMC5603500

[14] Zhang Y , Liu H , Li W , et al. CircRNA_100269 is downregulated in gastric cancer and suppresses tumor cell growth by targeting miR‐630. Aging (Albany NY). 2017;9(6):1585‐1594. 28657541 10.18632/aging.101254 PMC5509457

[15] Ding L , Ren J , Zhang D , et al. The TLR3 Agonist Inhibit Drug Efflux and Sequentially Consolidates Low‐Dose Cisplatin‐Based Chemoimmunotherapy while Reducing Side Effects. Mol Cancer Ther. 2017;16(6):1068‐1079. 28138030 10.1158/1535-7163.MCT-16-0454

[16] Zhou H , Rigoutsos I . The emerging roles of GPRC5A in diseases. Oncoscience. 2014;1(12):765‐776. 25621293 10.18632/oncoscience.104 PMC4303886

[17] Fabian MR , Sonenberg N , Filipowicz W . Regulation of mRNA translation and stability by microRNAs. Annu Rev Biochem. 2010;79:351‐379. 20533884 10.1146/annurev-biochem-060308-103103

[18] Zhao ZJ , Shen J . Circular RNA participates in the carcinogenesis and the malignant behavior of cancer. RNA Biol. 2017;14(5):514‐521. 26649774 10.1080/15476286.2015.1122162 PMC5449088

[19] Xia W , Qiu M , Chen R , et al. Circular RNA hsa_circ_0067934 is upregulated in esophageal squamous cell carcinoma and promoted proliferation. Sci Rep. 2016;6:35576. 27752108 10.1038/srep35576 PMC5067712

[20] Xu L , Zhang M , Zheng X , Yi P , Lan C , Xu M . The circular RNA ciRS‐7 (Cdr1as) acts as a risk factor of hepatic microvascular invasion in hepatocellular carcinoma. J Cancer Res Clin Oncol. 2017;143(1):17‐27. 27614453 10.1007/s00432-016-2256-7 PMC11819007

[21] Li P , Chen S , Chen H , et al. Using circular RNA as a novel type of biomarker in the screening of gastric cancer. Clin Chim Acta. 2015;444:132‐136. 25689795 10.1016/j.cca.2015.02.018

[22] Yoon JH , Abdelmohsen K , Gorospe M . Functional interactions among microRNAs and long noncoding RNAs. Semin Cell Dev Biol. 2014;34:9‐14. 24965208 10.1016/j.semcdb.2014.05.015 PMC4163095

[23] Piwecka M , Glazar P , Hernandez‐Miranda LR , et al. Loss of a mammalian circular RNA locus causes miRNA deregulation and affects brain function. Science. 2017;357(6357):eaam8526. 28798046 10.1126/science.aam8526

[24] Liu Q , Zhang X , Hu X , et al. Circular RNA related to the chondrocyte ECM regulates MMP13 expression by functioning as a MiR‐136 'Sponge' in human cartilage degradation. Sci Rep. 2016;6:22572. 26931159 10.1038/srep22572 PMC4773870

[25] Zhong S , Yin H , Liao Y , et al. Lung Tumor Suppressor GPRC5A Binds EGFR and Restrains Its Effector Signaling. Cancer Res. 2015;75(9):1801‐1814. 25744720 10.1158/0008-5472.CAN-14-2005

[26] Zougman A , Hutchins GG , Cairns DA , et al. Retinoic acid‐induced protein 3: identification and characterisation of a novel prognostic colon cancer biomarker. Eur J Cancer. 2013;49(2):531‐539. 23021913 10.1016/j.ejca.2012.07.031

